# Towards an integrated animal health surveillance system in Tanzania: making better use of existing and potential data sources for early warning surveillance

**DOI:** 10.1186/s12917-021-02789-x

**Published:** 2021-03-06

**Authors:** Janeth George, Barbara Häsler, Erick Komba, Calvin Sindato, Mark Rweyemamu, James Mlangwa

**Affiliations:** 1 Department of Veterinary Medicine and Public Health, Sokoine University of Agriculture, P.O. Box 3021, Morogoro, Tanzania; 2 SACIDS Foundation for One Health, Sokoine University of Agriculture, P.O. Box 3297, Morogoro, Tanzania; 3 Department of Pathobiology and Population Sciences, Veterinary Epidemiology, Economics, and Public Health Group, Royal Veterinary College, Hawkshead Lane, North Mymms, Hatfield, Hertfordshire, AL9 7TA UK; 4 National Institute for Medical Research, Tabora Research Centre, Tabora, Tanzania

**Keywords:** Surveillance, Animal health, Data source, Integration, Early warning, Tanzania

## Abstract

**Background:**

Effective animal health surveillance systems require reliable, high-quality, and timely data for decision making. In Tanzania, the animal health surveillance system has been relying on a few data sources, which suffer from delays in reporting, underreporting, and high cost of data collection and transmission. The integration of data from multiple sources can enhance early detection and response to animal diseases and facilitate the early control of outbreaks. This study aimed to identify and assess existing and potential data sources for the animal health surveillance system in Tanzania and how they can be better used for early warning surveillance. The study used a mixed-method design to identify and assess data sources. Data were collected through document reviews, internet search, cross-sectional survey, key informant interviews, site visits, and non-participant observation. The assessment was done using pre-defined criteria.

**Results:**

A total of 13 data sources were identified and assessed. Most surveillance data came from livestock farmers, slaughter facilities, and livestock markets; while animal dip sites were the least used sources. Commercial farms and veterinary shops, electronic surveillance tools like AfyaData and Event Mobile Application (EMA-i) and information systems such as the Tanzania National Livestock Identification and Traceability System (TANLITS) and Agricultural Routine Data System (ARDS) show potential to generate relevant data for the national animal health surveillance system. The common variables found across most sources were: the name of the place (12/13), animal type/species (12/13), syndromes (10/13) and number of affected animals (8/13). The majority of the sources had good surveillance data contents and were accessible with medium to maximum spatial coverage. However, there was significant variation in terms of data frequency, accuracy and cost. There were limited integration and coordination of data flow from the identified sources with minimum to non-existing automated data entry and transmission.

**Conclusion:**

The study demonstrated how the available data sources have great potential for early warning surveillance in Tanzania. Both existing and potential data sources had complementary strengths and weaknesses; a multi-source surveillance system would be best placed to harness these different strengths.

**Supplementary Information:**

The online version contains supplementary material available at 10.1186/s12917-021-02789-x.

## Background

In recent years, there have been increased concerns on the spread of animal infectious diseases due to their overwhelming impact on animal welfare [1], international trade [2], public health, ecosystem health and economic well-being of people who depend on animals as a source of livelihood [3, 4]. These overwhelming challenges highlight the need for more effective and efficient animal health surveillance systems. Animal health surveillance is the systematic, continuous or repeated, measurement, collection, collation, analysis, interpretation and timely dissemination of animal health and welfare-related data from defined populations [5]. Effective animal health surveillance systems depend on reliable and fit-for-purpose data sources, among other factors. Early warning surveillance systems must provide timely data and be sensitive in capturing and analysing any abnormal patterns to fast track the epidemiological investigation and allow appropriate actions to be taken [6]. Efficient animal health surveillance systems allow detecting animal diseases, including those transmissible between animals and humans (zoonoses) and informing control efforts [7]. Surveillance data can be used to estimate the magnitude of specific problems, determine the distribution of illness, portray the natural history of a disease, generate hypotheses, stimulate research, evaluate control measures, monitor changes, and facilitate planning [8].

The term “data source” represents a wide array of materials, from reports collected using an informal data collection process to sources that are a result of statistically designed, regular data collection processes that guarantee a high standard of quality [9]. Surveillance data may come from animal production units, veterinary clinical data, and livestock market surveillance [10],  laboratory diagnostics, sentinel surveillance, registries, surveys/research, administrative data systems [9], and electronic medical records [11]. Other sources of surveillance data include meat inspection reports and databases [12, 13], media sources such as PROMED-mail [13] and medicine prescription from the veterinary drug shops [14]. The selection of data sources must consider the content of data, spatial coverage, accessibility, data collection frequency, accuracy and cost [9].

The integration of data from multiple sources can provide a complete picture of the disease in the population by reflecting different aspects of the disease [15, 16]. For example, various data sources combined effectively may generate better information, thereby enhancing early detection of, and response to animal diseases  (e.g. facilitating the control of outbreaks). The integration helps to produce new data such as the relationship between cases reported, which may not be known by relying on a single source [17]. For instance, reported deaths of animals due to unknown cause in the farmers’ herds may be linked and compared to abattoir reports to help detect and control a potential epidemic. However, data from multiple sources may be heterogeneous, and of differing quality; hence their integration in a surveillance system may be challenging. Consequently, the added value of such integration will need to be compared to the resources required for establishing and running the system. A large amount of data and data sources can cause enormous integration cost [18] while low-quality data can deteriorate the quality of integration results instead of bringing the desired quality gain [19]. Inconsistency in the collection or processing may also limit the use of data from multiple sources [20].

Tanzania’s animal health surveillance is under the custody of the Directorate of Veterinary Service (DVS) through the Epidemiology Unit in the Ministry of Livestock and Fisheries (MoLF). The official reporting ladder for the livestock starts from livestock field officers to district veterinary officers (DVOs), zonal veterinary centres (ZVCs) to the DVS [20, 21]. Disease reporting is mostly passive, and clinical observation is the primary source of data. The collected data are filled in the designated paper-based field investigation forms and transmitted to the higher authorities weekly, either physically or through emails [20]. For notifiable diseases, a suspected case is reported to the DVO within 24 hours. The DVO visits the farm to investigate the case and report to the Officer Incharge of ZVC who in collaboration with the zonal Tanzania Veterinary Laboratory Agency (TVLA) will collect relevant samples. The ZVC will then notify the disease event to the DVS who has a mandate to transmit information internally and internationally. For wildlife, disease information may be captured by a veterinarian or game officer in the respective institutions such as the Tanzania Wildlife Research Institute (TAWIRI), Ngorongoro Conservation Area Authority and Tanzania National Park Authority (TANAPA) and information flow shall be through the respective DVO [21]. Despite these well-established structures, there are shortcomings that limit performance including delays in reporting and detection of animal health events, underreporting, high cost of data collection and transmission, lack of feedback and responses and limited infrastructures to support data flow from communities to the district [21]. To address these challenges, the MoLF designed a 5-year (2019–2024) animal health surveillance strategy outlining the theory of change for improving the surveillance system. The strategy pinpointed nine strategic issues to address, including promoting the use of real-time technology in surveillance. It also emphasized the improvement of data capture from various sources using innovative technologies as well as interoperability between existing information systems and information management tools. So far, the system has been relying on data from livestock farmers, slaughter facilities, livestock markets and zoo-sanitary checkpoints as the primary sources [21] with limited integration. To the authors’ knowledge, there is no systematically collected information on how best to utilize the existing data sources and to exploit untapped ones for early warning surveillance in Tanzania.

The study aimed to identify and assess existing and potential data sources for the animal health surveillance system in Tanzania and how they can be better used for early outbreak detection. This study formed part of the bigger research project titled “Prototype for cost-effective integration of animal health surveillance systems in Tanzania using systems thinking” that focused on how best to integrate the existing animal health surveillance systems by looking at all components of the system as a whole and their feedback loops including technical, social, financial, political and institutional aspects. The findings of this study provide inputs for the development of the prototype for surveillance systems integration and give insights into the implementation of the national animal health surveillance strategy, especially on data collection, management and decision making for early outbreak detection.

## Methods

### Study design

**Fig. 1:**
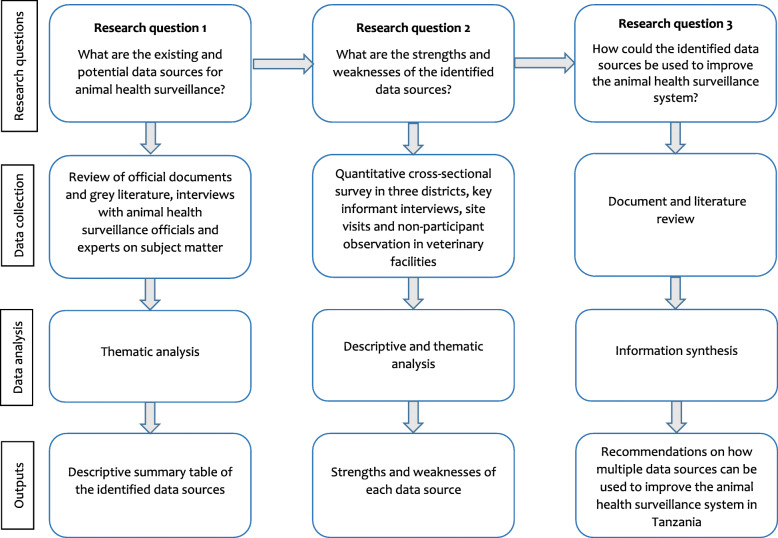
Research study process

The study used a mixed-method design to identify and assess existing and potential data sources. Figure 1 illustrates the study processes, including research questions, respective data collection methods, analysis and outputs.

### Identification and assessment of surveillance data sources

Data sources were identified from the review of animal health surveillance documents obtained through personal communication with officials of the relevant institutions, websites, grey literature search as well as interviews with experts on the subject matter and individuals from institutions working on animal health surveillance. Documents reviewed on animal health surveillance in Tanzania included: The Animal Disease Act 2003, the Veterinary Act 2003, the National Livestock Policy 2006, documents and implementation reports on the information systems and electronic surveillance tools. Additional literature was obtained through Google Scholar pages using the following search terms: “data sources” AND “animal health surveillance” OR “animal disease surveillance”. A total of 274 articles and reports were extracted but only 18 were relevant for this study. The literature obtained included research papers, reports and journal articles. All documents collated were read in full and used to extract information on data sources. The identified data sources were grouped into two categories: *Primary sources* which included data collected or generated directly for the national animal health surveillance and *secondary sources,* which included information collected for other purposes, with potential benefit to the national surveillance system. For both primary and secondary sources, information on the following variables was extracted: information collected/available on the source, frequency of data collection, data transmission modes, usage of information as surveillance data, as well as integration and its process.

Next, each data source was qualitatively assessed against the following six criteria: (i) data contents, (ii) spatial coverage, (iii) data collection frequency, (iv) data accuracy, (v) cost (is it free or are there charges involved), and (vi) data accessibility [9].

### Study setting

The study involved government and private institutions and veterinary facilities at national and district levels. At the national level, seven institutions and six veterinary facilities were conveniently sampled and visited. The institutions included the MoLF, TVLA, TAWIRI, Sokoine University of Agriculture-SACIDS Foundation for One Health and three ZVCs. The visited veterinary facilities included three zoo-sanitary checkpoints, two private poultry farms, and a cattle ranch.

**Fig. 2:**
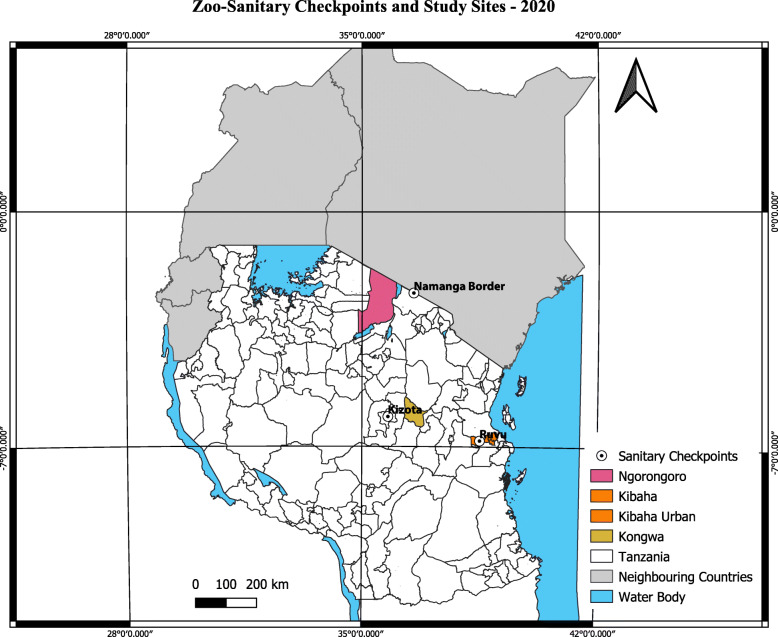
Map of the study districts and zoo-sanitary checkpoints (Personal creation using QGIS version 3.12.3-Bucureşti [25])

Three districts were involved in the study (Fig. 2). The purposive selection of the areas considered livestock production systems, location and cross-border interaction and level of surveillance intervention activities in the area. The selected districts were Ngorongoro, Kibaha and Kongwa. Ngorongoro was chosen because of high pastoral activities as it harbours a large number of pastoralists in a unique human-livestock-wildlife interface. Its closeness to a bordering country (Kenya) was an excellent opportunity to observe cross-border activities related to surveillance and ongoing intervention activities on improvement of human and animal health surveillance systems through mobile technologies. Kibaha is a peri-urban district with mixed livestock production systems, and it was included to observe whether proximity to the city (Dar es Salaam) influences the operationalization of surveillance systems. The district has also received some interventions on the improvement of the surveillance system. Kongwa is characterized by high pastoral activities, including national ranches. It has received minimum interventions in terms of surveillance improvement hence served as a comparative group in terms of data flow and response [21]. From the districts, 10 administrative divisions (hereafter called “wards”) were randomly selected using a random number generator from a list obtained from district economic profile reports and census data [22–24]. Veterinary shops, slaughter facilities, dip sites and livestock markets found in the selected wards were surveyed.

### Selection of participants

Participant selection combined random and purposive sampling methods based on the role they play in animal health surveillance and designations at the institutions. At the ward level, respondents were livestock field officers, people in charge of the public veterinary facilities and veterinary shopkeepers. At the district level, district veterinary officers or district livestock officers and livestock officers in charge of district-level veterinary facilities such as slaughterhouses and livestock markets were interviewed. At the zonal level, respondents were officers in charge of ZVCs and livestock officers at zoo-sanitary checkpoints. At the national level, the interviews were conducted with senior officials responsible for animal health surveillance system in the epidemiology unit, and Tanzania National Livestock Identification and Traceability System (TANLITS). We also interviewed people in charge of commercial farms, and animal health-related information systems and data managers from TVLA, SACIDS Foundation for One Health and TAWIRI.

### Data collection

#### Questionnaire administration

Two sets of structured questionnaires for the quantitative cross-sectional survey were prepared; one for government officials (livestock field officers and district veterinary officers) and another for veterinary shop owners/shopkeepers (supplementary files 1 and 2). The structured questionnaires were uploaded into the Open Data Kit (ODK), pilot tested with five livestock field officers and two veterinary shops in Morogoro municipal, refined using the information from the pilot testing and finally administered through face-to-face interviews. They included both open and closed questions. For government officials, the following items were included: primary sources of surveillance data, data collection procedures, data collection tools, frequency of data collected from the identified sources, data transmission, data management, and cost of data collection from the sources and transmission to the next level of authority. For veterinary shop owners/shopkeepers, the following questions were included: the average number of customers served per week, questions asked to the customer before dispensing the medicine, and record-keeping practices.

#### Key informant interviews

Semi-structured interviews were conducted with district veterinary officers, in charge of veterinary facilities and zoo-sanitary checkpoints. Others included community animal health workers, people in charge of commercial livestock farms, systems and data managers in the surveillance-related institutions and government officials responsible for animal health surveillance and related systems. The focus of the interviews was on the data generated and collected from the sources in their designated areas, data management, standard operating procedures, the status of the facilities and workforce, and triangulation of some of the data collected through other methods.

#### Site visits and non-participant observation

These methods were used to assess the physical conditions of the data source, observe the practices on the sites to compare them with the standard operating procedures and triangulate data obtained through other methods. Slaughter facilities, livestock markets, cattle dipping sites, zoo-sanitary checkpoints and commercial livestock farms were visited. The purpose of the observation was to take note  carefully of the practices and behaviour patterns of participants on the site without interfering in their routine activities. During the observation, the researcher (first author) found an inconspicuous spot with no involvement in any ongoing events and wrote down notes on the scenes in real-time. The researcher informed the participants about the reasons for their presence so that they did not change their practices and sometimes had post-observation informal interviews with some of them to clarify what was happening and why. The observation period was 1–4 h, and field notes were taken for later analysis.

### Data analysis

Data from questionnaires were downloaded into Microsoft™ Excel, cleaned and exported to IBM SPSS Statistics for Windows, Version 20.0 for descriptive statistical analysis using frequency and simple percentage. Data from the observations were summarized manually in Microsoft Word. Interview data were transcribed, reviewed and manually coded in MS Word. Similar responses were extracted and grouped into clusters of themes and then analysed using deductive thematic analysis to establish the patterns of the findings. Documents reviewed were analysed by using content analysis method [26, 27]. The two sets of data (qualitative and quantitative) were combined in the analysis of results for interpretation. Information on the strengths and weaknesses of each data source obtained through interviews, observation and document reviews were provided in tabular form. The data sources were ranked as maximum, medium and minimum for each criterion based on the descriptors provided in Table 1. The blank cell means no data were available for this criterion.

**Table 1:** Data source assessment criteria and descriptors

Criterion		Descriptors		
	Descriptive question	Maximum	Medium	Minimum
Data content	Does the source contain relevant surveillance data for analysis and decision making?	It contains all relevant surveillance variables	It contains five or more variables which can be used for analysis	It contains less than five variables which can be used for analysis
Spatial coverage	What is the spatial coverage of the data source?	National level	Area-specific but covers more than one district	Specific to one district
Data collection frequency	How frequent are data collected and transmitted from this source?	Weekly or monthly	Six months or annually	No exact data collection schedule or data have never been collected
Data accuracy	Are the processes and data collection methods clear to guarantee data completeness and provenance?	Full and comprehensive documentation of the data collection process	Partial documentation of data collection process	Almost no information available on data collection process
Cost of data	Are there costs associated with data collection and transmission from the source?	No cost associated with data collection and transmission	Yes, costs are covered by the recipient of data.	Yes, costs are covered by the person who collects and physically transmits data.
Data access	Are the data drawn from this source routinely available for surveillance-related analysis?	Data are readily available, organized and can be accessed without strict procedures	Data are available but are not routinely collected and organized	Data are rarely or never collected from this source

## Results

### Overview of the identified data sources

Identified existing surveillance data sources were: (i) Livestock owners, (ii) Veterinary professionals, (iii) Veterinary facilities including slaughter facilities, animal dip sites, livestock markets and zoo-sanitary checkpoints, (iv) Information systems such as laboratory information management system for TVLA (*Sistema Informativo di Laboratorio:* SILAB), TANLITS, Agricultural Routine Data System (ARDS) and (v) Databases of electronic surveillance tools such as AfyaData and Event Mobile Application (EMA-i). Potential data sources identified were veterinary shops and commercial livestock farms.

**Fig. 3:**
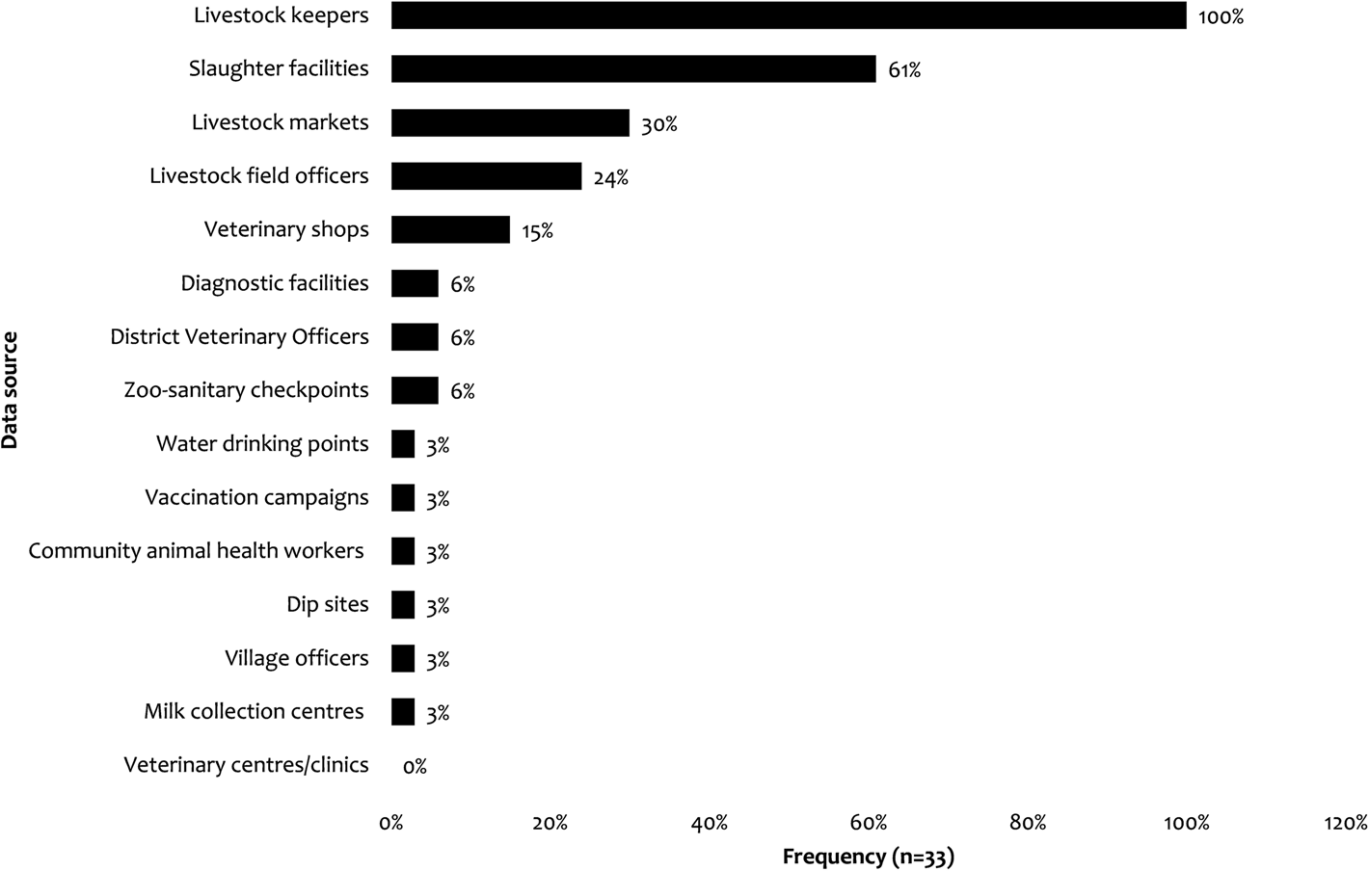
Sources of animal health surveillance data at the district level (*n, is the number of questionnaire respondents)*

Table 2: Description of the identified data sources. Through questionnaires administered to 33 respondents at the district level, it was found that data were mainly sourced from livestock farmers (100%), slaughter facilities (61%), and livestock markets (30%) (Fig. 3). Apart from livestock farmers, slaughter facilities were frequently used in Kongwa (44%, *n* = 18) while livestock market data collection was a common practice in Ngorongoro (40%, *n* = 10) and veterinary shops were reported more frequently in Kibaha. Other sources included vaccination campaigns, animal water drinking points, and community animal health workers.

**Table 2:** Description of the identified data sources

Data source	Surveillance purpose	Activity	Output	Coverage	Source category
Livestock farmers	Disease control, animal health management and production	Reports any affected, suspected or died animal of any disease or from any cause	Information on the affected animals such as species, age, sex, number of animals affected and symptoms	National	Primary
Slaughter facilities	Disease control, public health, food hygiene and safety	Ante-mortem and post-mortem inspection of animals brought to the facility	Inspection reports on number and species of animals slaughtered, number and species of animals condemned (whole or parts) and reasons for condemnation in that particular facility	National	Primary
Animal dip sites	Control of parasitic diseases such as tick-borne diseases.	Regular dipping of animals into the dip tanks	Reports on number and species of animals dipped in that particular facility	National	Primary
Livestock markets	Disease control and movement tracing	Screening of animals for diseases and issuing of movement permits at the markets	Reports on number and species of arrived, number and species of animals sold and clinical signs observed	National	Primary
Zoo-sanitary checkpoints	Disease control, animal welfare and movement tracing	Inspection of all animal trucks, verification of documents and physical inspection of the animals for disease screening and injury Quarantine of disease suspected consignments	Report on the number of vehicles arrived and number and species of animals aboard, clinical signs or injuries observed	National	Primary
Veterinary shops	Selling of drugs and veterinary equipments	Advice and drug dispensing to livestock farmers	Data on the most reported symptoms	National	Primary
Commercial livestock farms	Disease control, animal health management and production	Reports any affected, suspected or died animal of any disease or from any cause	Data on the number of animals affected by a disease treated and vaccinated	Area-specific	Primary
SILAB	Tracking of the sample from the point of collection to result	Tests of biological samples from zonal veterinary centres (ZVCs) and private clients	Test reports to customers and DVS if a notifiable disease	National	Secondary
TANLITS	Animal identification and registration of movements and other health-related events	Registration, identification and tracking using group and unique identification	The IDs which can be used for animal health surveillance	National	Secondary
Agricultural Routine Data system (ARDS)	Official data collection system in the agricultural sector	Collection, management, and transmission of agricultural performance information from local government authorities to Agricultural Sector Lead Ministries	Reports on the affected animals such as species, age, sex, number of animals affected and clinical signs	National	Secondary
AfyaData database	Enhanced syndromic surveillance covering human, animals and environment	Geo-referenced syndromic data collection from the community level, real-time data transmission, data analysis and visualization and feedback	Surveillance reports on the submitted data and feedback to the reporter	Area-specific	Secondary
EMA-I database	Enhanced animal disease reporting and decision-making support	Collection of geo-referenced data from the district level, real-time data transmission to Global animal information system (EMPRES-i), data analysis, and visualization.	Surveillance reports on the submitted data	Area-specific	Secondary
TAWIRI	Wildlife health management and disease control	Test biological samples from wildlife and livestock in the interfaces	Test reports and to DVS if a notifiable disease	Area-specific	Secondary

### Data flow and management from different sources

**Fig. 4:**
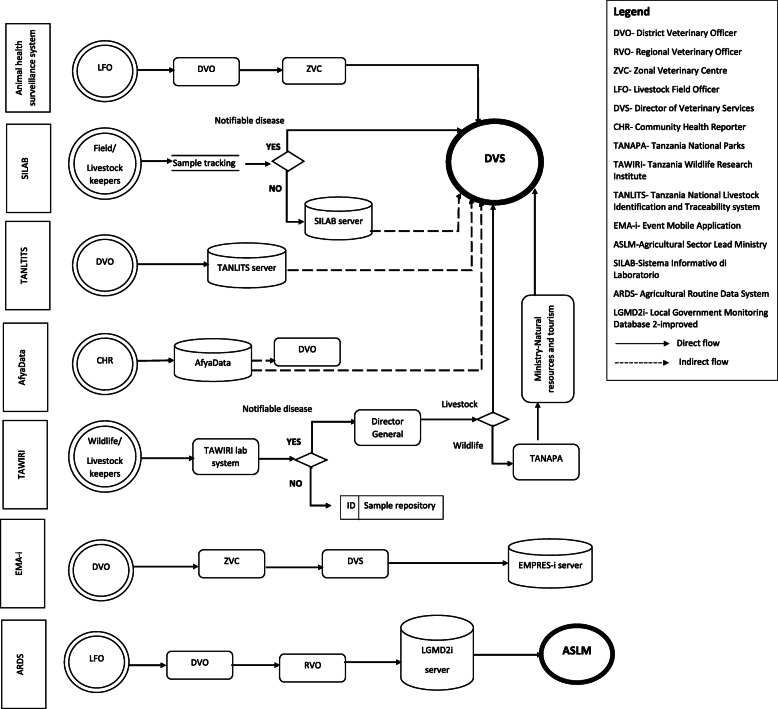
Current flow and management of data from different data streams

Figure 4 illustrates the current data flow and management in the mainstream and other systems that contain surveillance data. In the national animal health surveillance system, data collected from the identified sources are transmitted from livestock field officers as the first line of communication to district veterinary officers for compilation and verification. Afterwards, the data are submitted to the ZVC, where they are compiled and verified and then submitted to the MoLF. The ministry compiles data from all the zonal veterinary centres in a database. Zoo-sanitary checkpoint data are sent to the ZVC for compilation and verification before being forwarded to the MoLF. SILAB and TAWIRI only report notifiable cases while retaining the rest of the sample data. Data from EMA-i goes to EMPRES-i at FAO. AfyaData data are stored in a server which is located at the SACIDS Foundation for One Health, at Sokoine University of Agriculture for research purposes but can also be accessed by the DVOs for weekly and monthly reporting to the DVS. For the TANLITS, information about a particular animal is entered into the system by the DVO and reflected in the database at the MoLF. Some data are collected and channelled into the ARDS. The collected data are transmitted to the district livestock/veterinary officer in hardcopies, entered into the system, and sent directly to the server, which is under the Ministry of Agriculture and Food Security.

## Current status, strengths and weaknesses of the identified data sources

### Existing data sources for the national animal health surveillance system

#### Livestock farmers

Livestock farmers were the main source of surveillance information which can be captured when they seek veterinary services or reported by livestock field officers recorded during routine farm visits for clinical services, vaccination and reproductive service (Fig. 3). About two-thirds (68%, *n* = 28) of livestock field officers collected data from the farmers daily, and on average they spent 20 to 90% (median = 65%) of their working time attending to animals. Twenty-nine per cent of the respondents reported that livestock farmers reach out to them upon suspecting cases. During the observation at the livestock markets, it was also learned that some farmers bought stocks of veterinary medicines from street vendors for self-treatment of their animals, which may imply that they only report to the veterinary professional when the case had worsened.

#### Livestock markets

Three livestock markets were visited for non-participant observation. At the markets, the procedure requires an inspection for legal documentation and screening of animals for any disease symptoms and issuance of movement permits after the auction. It was found that livestock markets were holding a large number of animals from different places, including outside the district. From June to August 2019, a total of 45,668 sheep and goats and 14,456 cattle were sold in 13 livestock markets in the study districts. The distribution of livestock sold per district and month is summarized in Table 3. The data collected at the markets included the name of the custodian and the number of animals brought for sale, number and species of animals screened and number and species of animals sold. Only one market was screening for diseases at the entry point. After the auction, only animals that were going outside the village were granted a movement permit at the loading area or exit gate. Animals which remained in the village where the market is, did not receive any document apart from levy fee receipt.

**Table 3:** Number of livestock sold by districts between June and August 2019

Month	June		July		August	
District	Goat and sheep	Cattle	Goat and sheep	Cattle	Goat and sheep	Cattle
Kibaha (*n* = 2)	52	146	53	173	44	225
Kongwa (*n* = 1)	1205	402	1328	443	1081	360
Ngorongoro (*n* = 10)	14,511	4347	13,972	4274	13,422	4086

(n, represents the number of livestock markets from which data were extracted)

Source: District Livestock Offices

#### Slaughter facilities

Data from slaughter facilities are communicated to the DVO through a monthly report or within 24 h for notifiable diseases. The report contains information about species, condemned organs/animal carcass (partial or total), reasons for condemnation, and the number of condemned animals due to that particular reason. A total of 20 slaughter facilities were visited. They slaughter between one and 20 livestock per day. The majority of the facilities were slaughter slabs (85%, *n* = 20) with no proper infrastructures such as lairage areas (holding pens) and water and drainage systems. Only five facilities were doing ante-mortem inspection a day before slaughter due to lack of lairage areas and security in most of the facilities.

#### Zoo-sanitary checkpoints

This is another official surveillance data source. Three zoo sanitary checkpoints were visited. On average they attended 1–4 vehicles per day which carry 25 head of cattle or 250 goats/sheep each. All the checkpoints were actively checking all the animal trucks, cross-checking, and verifying all the documents and physical inspection of animals aboard for any clinical signs or injury. At the border post, animals that cross to the neighbouring country were issued animal health export certificate indicating port of exit and destination, reasons for exportation, disease status, and control measures which are taken in the country of origin. Despite the active involvement in surveillance and observation of animal welfare, zoo-sanitary checkpoints were highly constrained of human resources and infrastructures. Each of the visited checkpoints had only one livestock officer who did everything on-site and who only worked during the day yet some trucks pass during the night. Only one of the checkpoints had most of the required infrastructures such as holding grounds, loading and offloading bays and water troughs while others were lacking such infrastructure. Other mentioned and observed challenges were paper-based record keeping, some truck drivers skipping checkpoints, and lack of unique identification (ID) for animals that make it hard to verify the consignments.

#### Dip sites

**Fig. 5:**
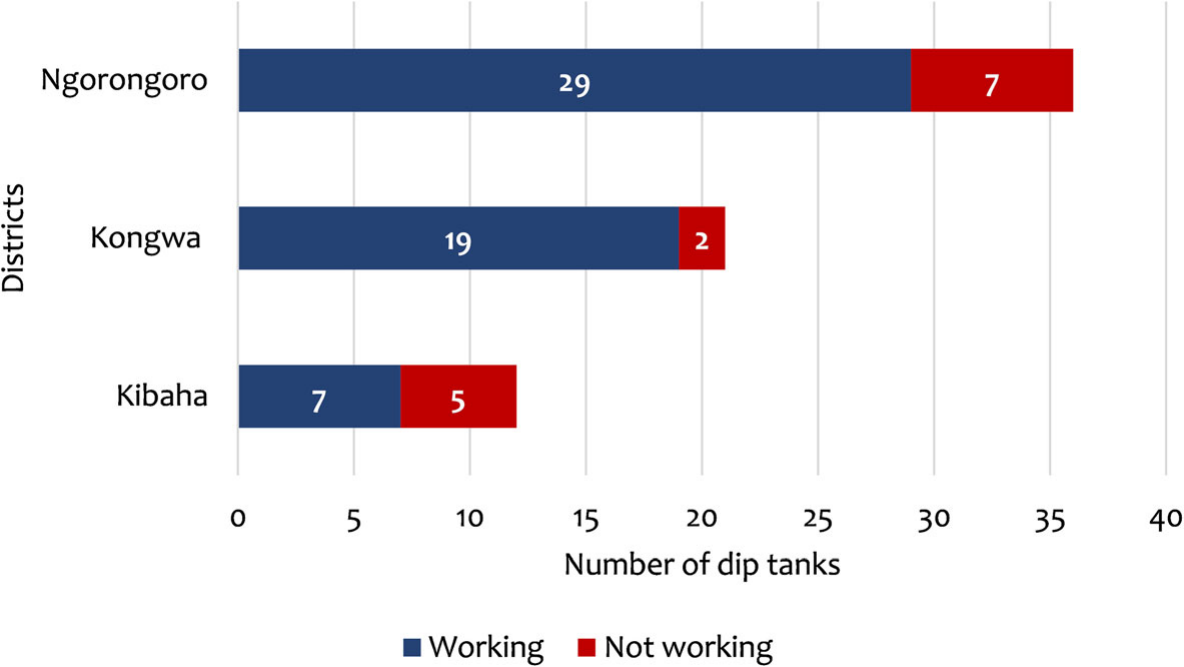
Distribution of dip tanks by district

This was among the least used sources of data despite been recognized as an official source. In the study districts, there were 69 dip tanks (80% were working). Ngorongoro had the largest number of dip tanks while Kibaha had the least (Fig. 5). It was also noted that 58% of the dip tanks in Kibaha were privately owned while in other districts were either public or communally managed. Thirteen public dip sites were visited, and five of them were working, and they were mostly dipping cattle. On average 1500–6000 (media*n* = 2258) animals were dipped monthly or bi-weekly. The frequency of dipping was said to increase during the tick season. Farmers who used the service had to pay about TZS 100–200 (US$ 0.04–0.09) per animal for the upkeep of the dip tanks and purchasing of acaricide. Information on the number of animals dipped was integrated into the monthly report and submitted to the higher authorities. It was observed that on the dipping days, a lot of animals were brought into one place and sometimes stayed the whole day waiting for the service. There was no screening of animals before entering into dip tanks. Also, it was reported that the usage of the sites was not consistent; not all the villages were actively using them while some farmers did not bring their animals for dipping. Some of the reasons for lower compliance were unaffordable fees, lack of dipping acaricides, and non-compliance by some  farmers, which demoralized others.

#### Laboratory information management system **(**Sistema Informativo di Laboratorio: SILAB)

This system is for tracking of the sample from the point of collection to result using unique identification number (ID). Samples from ZVCs or individual farmers were being processed in any of the 12 centres of TVLA distributed across the country. The processed samples contained details such as the origin of the sample, species and type of material, e.g. serum. Additional epidemiological data were also recorded, e.g. the number of animals affected. The test report is sent to the customer who requested the test, and in case of a notifiable disease, information is also sent to the DVS. The rest of the sample data are stored in the database. The major limitation of the system is the server being hosted outside the country which makes it challenging to customize.

#### Tanzania Wildlife Research Institute (TAWIRI)

This is a data source for wildlife surveillance. General surveillance between wildlife and livestock in the interface areas were being conducted yearly if no reported cases. The institute had its laboratory for sample processing and storage system. Notifiable disease in livestock get reported directly to the DVS while for wildlife, the reports were sent to TANAPA then to the ministry responsible for natural resources which will then forward the data to the DVS.

### Potential data sources for the national animal health surveillance system

#### Commercial livestock farms

Three commercial farms were visited; a poultry farm in Kibaha, a cattle ranch in Kongwa, and an animal breeding company in Arusha. All the farms reported to keep records of animals that fall sick, are vaccinated, and treated. Only notifiable diseases are reported to the respective government officers. One of the farms had an electronic ear tagging system to trace all the information about their livestock, and data were linked to electronic performance monitoring system through the ODK.

#### Veterinary medicine  shops

**Fig. 6:**
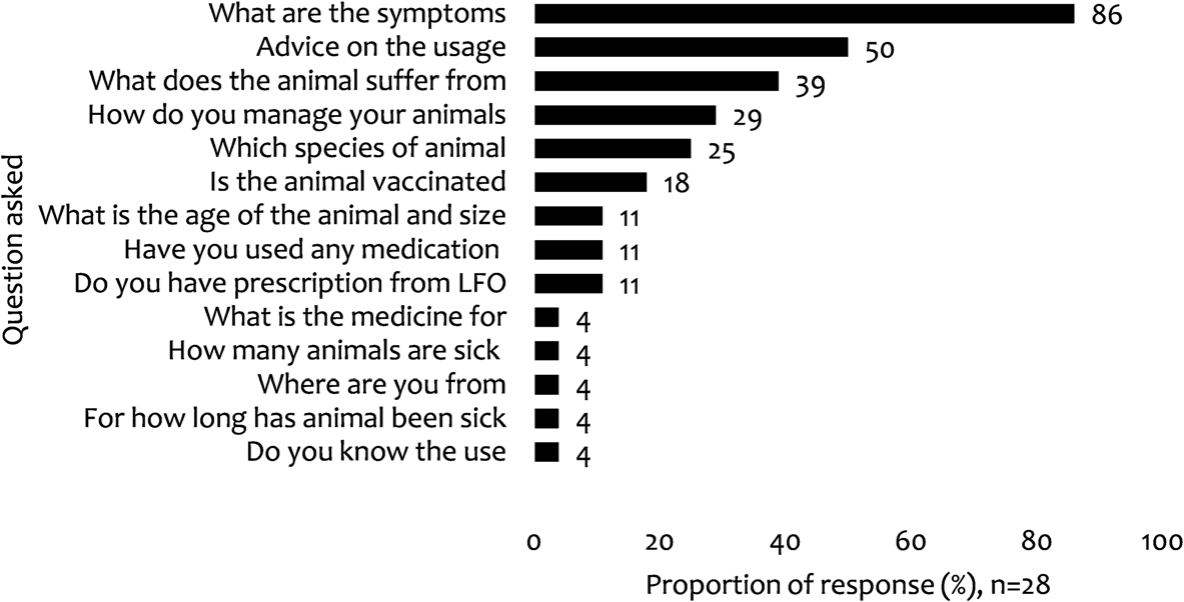
Frequently asked questions from veterinary shopkeepers to the clients

Twenty-eight veterinary medicine shops were visited in the three districts (Kongwa = 13, Ngorongoro = 8, Kibaha = 7). On average, one shop served between 8 and 720 (median = 72, SD = 195) clients per month. The average number of clients per month in each district were: Kibaha 120, Kongwa 40 and Ngorongoro 70. The majority of these shops were selling both plant protection and veterinary products. Before dispensing medicine, the respondents claimed to seek some information from the clients to ascertain their knowledge and skills in medicine use (Fig. 6). Commonly sought information included, symptoms manifested by the animals, the suspected disease and animal health management. Very few shopkeepers (11%, *n* = 28) asked for a prescription certificate from a veterinarian. They were keeping records of the medicine sold in hard copies for accounting and procurement purposes. Some shopkeepers did not have an animal health education background but used the acquired experience to prescribe and dispense drugs.

#### Tanzania National Livestock Identification and Traceability System (TANLITS)

Some of the variables recorded in the TANLITS include owner, premise (location), details of the animal (breed type and age), and veterinary services received (e.g. vaccination). The system contained both electronic and manual identification data. As of the year 2019, a total of 70,000 cattle were registered into the system using unique ID electronic ear tags and 17 million (cattle and donkeys) registered through group identification. The system is flexible to accommodate more variables such as livestock products which can be linked with particulars of the registered animal. The system has managed to register animals but does not include tracking information.

#### Agricultural Routine Data system (ARDS)

The ARDS  included both crop and livestock data which were collected from the farmers and submitted to the Ministry of Agriculture monthly. Some of the health-related data contained in the system are veterinary services offered (e.g. artificial insemination, castration, pregnancy diagnosis, etc.), dipping and spraying, and vaccination (number of animals and vaccines used). It also included meat inspection (species, number, condemnation, and reasons) and disease status report (species, number of affected animals, treated, died and recovered). Data collection is paper-based, and data entry into the system starts at the district level and forwarded to regional authorities for verification before submission to the ministry. Despite being a standard reporting tool among livestock officers, animal health-related data are not synchronized with the national animal health surveillance system (which is under the Ministry of Livestock). Data collection and transmission from the field were found to be costly since they have to submit physical copies to the district offices.

#### Data from electronic surveillance tools

AfyaData and Event mobile application (EMA-i) are electronic tools which have been used in the collection, real-time data transmission, analysis and transmission of surveillance data from the field using mobile phone technologies. AfyaData, which means “health data” in Kiswahili, is a One Health disease surveillance tool that was founded because of the need to identify infectious diseases in the early stages, in order to allow provision of information through collection, analysis, interpretation and real time transmission of data to be used to help with diagnosis and treatment. It was designed, developed and deployed collaboratively between animal health, human health, Information Communication and Technology, socio-anthropology experts and community members. The tool uses multiple languages, has a feedback mechanism and flexible in data storage and customization. One Health Knowledge Repository functionality is integrated into AfyaData application, which powers it for prediction of the most likely disease condition based on collected syndromic data. The design of AfyaData has been explained in detail by Karimuribo et al. [28, 29]. On the other hand, EMA-i was designed by FAO to facilitate data collection and real-time disease reporting from the field to the chief veterinary officers and serves as official reporting tool [30].

Apart from mobile applications, AfyaData and EMA-i have web-applications that serve  as data management platforms. The tools were designed to capture geo-referenced data (both online and offline) to enhance contact tracing and  spatial analysis of the reported incidences. The former collects syndromic data for human and animals from the community level, while the latter is used by the district veterinary officers to capture priority zoonotic diseases at the district level. EMA-i allows data verification and validation at each level before being submitted to the server. AfyaData has been used in five districts while EMA-i has been been rolled out in 74 districts of Tanzania. Some of the observed and reported limitations included the use of internet for data submission which come at a cost to the sender but also not all areas have internet coverage; therefore, the time between data capture and submission may not always be real-time. EMA-i may not be flexible and easy to customize to country-specific needs since its server is hosted outside Tanzania. Data verification and validation may mean additional time before data reaching the server. AfyaData users reported limited response after sending data. Table 4 provides the summary of strengths and weakenesses of each data source.

**Table 4:** Descriptive summary of strengths and weakness of each data source

Data source	Strengths	Weaknesses
Livestock farmers	Early warning for diseases with clear clinical signs Easy case detection due to high coverage	Not all farmers report the disease events due to factors such as the negative consequence of reporting or the value of the animal. Diseases with subclinical signs may not be reported.
Slaughter facilities	A constant supply of surveillance data because of high slaughtering frequency It is easy to collect specimens for laboratory diagnosis. Data collection is less costly.	Poor infrastructures such as lack of lairage areas (holding pens) hence making it difficult for ante-mortem inspection. The collected data is not directly sent to the Ministry of Livestock and Fisheries; instead, they are sent to the Ministry of Agriculture and Food Security through ARDS.
Animal dip sites	A large number of animals convened at one place during dipping It is easy for visual inspection and screening. It is less costly because data can be collected while waiting for dipping. High coverage because they are found all over the country	Not all farmers bring their livestock to the public dip tanks. Diseases with subclinical signs may not be reported.
Livestock markets	It is easy for visual inspection and screening. They bring a large number of animals from different places weekly or monthly. It is less costly because animals can be screened before entering the auction bay.	Screening of animals wasn’t consistency. Conflict of interest between surveillance and revenue collection at the markets A limited number of human resources for screening and visual inspection
Zoo-sanitary checkpoints	Data are readily available because every livestock consignment has to be pass-through identified checkpoints en route their destinations.	Incomplete data in some of the reports Most of the checkpoints are human resource-constrained No coordinated tracking system for the consignments in designated routes
Veterinary shops	They serve a large number of livestock farmers per month. They enquire symptoms and animal health management. High coverage because there is at least one shop in every ward	They are not well regulated. They only keep sales records and not about symptoms.
Commercial livestock farms	They have organized record keeping.	They only report notifiable diseases.
SILAB	The system is automated from sample collection to test report. High coverage as it operates in entire national laboratory network in the country	Data are not linked to the Ministry of Livestock and Fisheries except for notifiable diseases. The server is not hosted in the country hence make it challenging to customize variables. Sample processing is not free; hence not many people take samples for testing.
TANLITS	It keeps the register of all livestock in the country for identification and tracking. Dairy cattle have unique IDs. The system is flexible to accommodate more variables. GPS embedded features.	Groups identification stops at the village level hence makes it difficult to trace individual animals. Requires regular updates of the shapefiles due to constant changes in administrative boundaries.
Agricultural Routine Data system (ARDS)	Data collection is coordinated from lower to ministry level. Data are submitted monthly.	Data collection and transmission is still manual hence take a lot of time and prone to human errors. The collected data are not linked to the Ministry of Livestock and Fisheries. Data submission rate is still low
AfyaData database	Near real-time data transmission GPS embedded features Collects syndromic data Covers both animal and human health data Point of capture is at the community level.	Requires smart-phone technologies and must be connected to internet services
EMA-I database	Near real-time data transmission GPS embedded features Collects case-based data Data verification at various levels	It only records presumptive diagnosis; hence there are chances of missing new symptoms. Requires smart-phone technologies and must be connected to internet services The server is not hosted in the country; therefore, it may not be flexible to make changes in the variables.
TAWIRI	A well-coordinated sample collection system	Data collection is expensive and not regular. Only notifiable diseases are sent to DVS

### Convergence and divergence of data sources

There were some convergence and divergence in the information recorded in the identified data sources. The following records were found in at least one of the 13 data sources: name of the place, animal type/species, sex, age, geo-reference, case, clinical signs, photo, number of affected animals, number of recovered animals, number of deaths, number of treated animals and type of medication used for treatment, and diagnostic test conducted. The common variables found across most of the sources were the name of the place (12/13), animal type/species (12/13), syndromes (10/13) and the number of affected animals (8/13) (Additional file 1). Geo-referenced data were only captured through AfyaData, EMA-i and TANLTIS.

### Quality of the identified data sources

Table 5 presents a summary of the qualitative assessment of the data sources based on six pre-defined criteria. The results were ranked into maximum, medium, or minimum based on the available data. Overall, all the sources had surveillance data elements, but there were variations in the contents and other qualities. Only AfyaData and EMA-i contained exclusive surveillance data, while the rest had medium (9) to minimum (2) content. 7/13 (53.8%) of the data sources had national coverage while others were area-specific. For instance, most of the zoo-sanitary checkpoints and veterinary laboratories were at the regional and zonal levels while AfyaData and EMA-i tools were piloted in some of the districts. Data collection frequency and accuracy varied across the data sources. The cost of data could only be established in 8/13 sources, where 87.5% of them were medium. Data accessibility was mostly medium.

**Table 5:** Quality of the identified data sources

Data source	Data content	Spatial coverage	Data collection frequency	Data accuracy	Cost of data	Data access
Agricultural routine data system (ARDS)	◐	●	◐	◐	◐	●
Livestock keepers	◐	●	◐	◐		◐
Commercial livestock farms	◐	◐	◐	●		◐
Livestock markets	○	●	○	○		○
Animal dip sites	○	●				
Slaughter houses/slabs	◐	●	●	◐		●
Veterinary shops	◐	●	○	○	○	◐
Zoo-sanitary checkpoints	◐	◐	◐	◐	◐	◐
AfyaData	●	◐	●	●	◐	●
SILAB	◐	◐	◐	◐	◐	◐
EMA-i	●	◐	●	●	◐	●
TANLITS	◐	●	○	◐	◐	◐
TAWIRI	◐	◐	◐	●	◐	◐

Key: ●=Maximum, ◐= Medium, ○=Minimum, Blank = No data available

## Discussion

This study aimed to identify and assess existing and potential data sources for the animal health surveillance system in Tanzania and to establish how to make better use of them for early warning. A total of 13 data sources were identified and assessed. The results indicate that most data come from livestock farmers, slaughter facilities, and livestock markets, while animal dip sites were the least used sources. Commercial farms and veterinary medicne shops, electronic surveillance tools like EMA-i and AfyaData and information systems such as TANLITS and ARDS have unused potential to generate relevant data for the national animal health surveillance system. The majority of the sources had good surveillance data contents and were accessible with medium to maximum spatial coverage. However, there was significant variation in terms of data frequency, accuracy and cost.

All the identified data sources contained animal health-related data, which are of relevance to surveillance even though some were not actively collected. Structures such as livestock markets, slaughter facilities, dip tanks and zoo-sanitary checkpoints are already in place and receive a good number of animals per month. Livestock markets bring together a large number of animals from different locations every month and sometimes more than once a month. In Tanzania, there are 521 livestock markets scattered all over the country [21] thereby highlighting their potential usefulness for surveillance. Several studies have shown that livestock markets and trade networks can be hotspots for infectious diseases [31, 32]; thus surveillance in such places using at least visual inspection is likely to be of value [6]. Surveillance at the slaughter facilities allows the detection of disease conditions before the meat is passed into the human food chain. A post-mortem has the potential for easier detection of diseases which could not be captured through visual inspection of a live animal [13]. Veterinary shops and commercial livestock farms were not commonly used as data sources in the official reporting system, but they were found to contain relevant surveillance information. The former generates data through regular contact with livestock farmers who come for medicine and consultation while the latter has coordinated data collection procedures. Veterinary shops were found to serve more livestock farmers compared to livestock field officers. This observation is in line with what was reported in a study conducted by Onono et al. [33]. Some of the reasons for that pattern is partly attributed to the limited number of official animal health professionals [21], associated costs and established relationships and trust between livestock farmers and private veterinary service providers [34]. The shopkeepers are consulted, prescribe and dispense drugs based on the symptoms narrated by the client. They do not keep records of who they have served and for what, but it is straightforward to tell what kind of disease is circulating based on the reported symptoms.

There are already several data collection tools in place, both paper-based and electronic, as well as databases such as EMA-i and AfyaData. The use of these electronic tools provides an opportunity for improving the current reporting system through real-time transmission and geo-referenced data. Embedded GPS features in the applications make it easier to locate and visualize outbreak zones for immediate intervention. Nevertheless, the sustainability of the technology use is still a challenge due to limited access to internet services, and software and technology maintenance [35]. The initial cost of setting up the system and political commitment are also important driving factors for adoption of new  technologies.

Data sources containing similar variables can be used to signal any suspicious pattern of a health event before it turns into an outbreak and to estimate the impact of a disease that affects production. However, the study found limited coordination and integration of data flowing from these sources. Some of the institutional databases were not linked to the national epidemiology unit. Data flow from some of the sources were either partially or not automated at all, which means the transmission and entry from one level to another were manually done; this may be more time consuming and may affect the quality of data [36, 37]. Since the disease reporting is mostly passive, data frequency and consistency varied considerably across the sources. Data from livestock farmers were mainly through self-reporting or routine veterinary visits while slaughter facilities reported post-mortem data. Screening for diseases at the livestock markets and dipping sites was not a common practice. Zoo-sanitary checkpoints were found to be an active data source but were not well-coordinated and highly resource-constrained. Data flow from a particular source depends on the incentive behind reporting and the effort required to complete and submit reports or perform other surveillance tasks [37]. For instance, farmers’ reporting depends on the expected feedback and associated consequences such as penalty [38]. Some studies have also shown that a farmer’s decision to call a veterinarian considers the economic value of the animal, the effect of the disease, severity of the symptoms, availability of veterinary service, and previous experience in treating the disease [10, 39].

Efficient animal health surveillance system requires the selection of suitable data sources to ensure consistency and reliability, which will inform the decision-making process. The results indicate that none of the existing or potential data sources meets all the criteria fully, but they can contribute relevant data. Gan et al. [15] argue that a single data source is not sufficient in identifying all new cases, which could lead to a severe underestimation of the real burden of the disease in the population. Gates et al. [10], also warn that over-relying on veterinarian reports or laboratory confirmation of infection can lead to significant delays in detection, especially if farmers do not seek veterinary consultation for sick animals. Therefore, to make better use of data sources, there is a need for complementarity and trade-off between specificity and timeliness.

Incentivization of reporting, integration of animal health services, standardization and integration of data are among the proposed solutions for improving data generation, flow and use of data from multiple sources. Incentivization of reporting can be achieved through prompt and appropriate feedback [29], clinical assistance or financial compensation to those who report as well as clear communication on how the data they provide can be analysed and used [40, 41]. Integration of veterinary services at the dip sites may help to motivate more people to use the services while generating surveillance data through screening. Some of the services which can be offered in addition to dipping may include creating awareness on the epidemiology of various diseases, sensitization on acceptable animal health management practices, and consultation. The government can support these efforts through subsidizing of acaricides and promoting the accessibility of water.

Interoperability of information systems and electronic tools for surveillance purposes helps to ensure timely detection and response to disease epidemics. Integration of information systems may follow these steps: Data integration process, establishing a central database for data repository and dashboard, standardization of data formats and setting a threshold for each case [17, 42, 43]. Such integration allows detection of abnormality once the number of reported cases or syndromes exceed the threshold and send alerts to the respective authorities for actions. From there, the investigation team reviews the aberration to identify if there is a potential outbreak or new symptoms by checking if there is any unusual pattern. The team will also decide on the severity of the event by categorizing it into low, medium or severe depending on the outcome. If need be, they may call for an investigation or additional information from the source.

The use of mobile technologies for data collection and transmission from the sources will help to improve timeliness, reduce human error and level of effort for data entry and transmission along different data flow levels [36]. This may leverage on the mobile telecom service subscription and internet user penetration rate in Tanzania, which stand at 48,056,689 and 46% respectively as of June 2020 [44]. The existing electronic surveillance tools which already have important features and databases in place will help to increase data accessibility, timeliness and simplify outbreak detection through GPS coordinates. Enhancing interoperability between AfyaData and EMA-I, with appropriate customization offers two additional benefits: (i) the prospect of a shared One Health surveillance platform that can be duly customized for human health and animal health as well as automatic authorized information exchange between the two sectors, especially in respect of zoonoses; (ii) the potential for community to global level data flow, subject to appropriate gating and authorization. Given the current configurations, EMA-i can be extended to livestock field officers as the point of capture for case-based reporting. At the same time, AfyaData could be used by veterinary shops and individual farmers for syndromic data.

Institutional arrangements and structures are among the critical elements of the integration process. For the integration to be operational, there is a need for coordination among institutions that take part in surveillance. Institutional arrangements are the policies, practices and systems that allow effective functioning of an organization or groups which may involve both hard and soft rules [45]. The existing information management systems such as TANLITS, ARDS and SILAB and databases for AfyaData and EMA-i are hosted in different institutions, and do not speak to each other despite having common variables. For integration to be feasible, the participating institutions will need to agree on the modality of interoperability, standardization and data management and security. These arrangements will only be possible by promoting inter-institutional relations and interactions.

To make better use of multiple data sources, several studies have demonstrated how their integration could improve health surveillance systems’ performance, including sensitivity, timeliness, and data quality [42, 46–48]. For the integration to be operational, policymakers and surveillance actors may work towards: (1) Establishing multi-source central data repository (2) Digitalization of data collection using electronic tools, (3) interoperability of the animal health systems and platforms which are TANLITS, ARDS, SILAB, AfyaData and EMA-I and (4) standardization of data. To achieve this, it will be essential to develop a data-sharing policy between participating institutions, integrating veterinary services such as dipping, vaccination and animal health management, and active community engagement, including participatory surveillance. In the next step of the programme, the economic value of various integration options will be investigated to identify the most cost-effective integration mechanisms. Finally, recommendations for a prototype systems integration will be established by combining information on the value of using multiple data sources, potential integration mechanisms, communication and institutional arrangement and economic analysis of integration to support the operationalization of a more effective and integrated surveillance system in Tanzania.

## Conclusion

The study has demonstrated how the available data sources have great potential for improving animal health surveillance system in Tanzania. Both existing and potential data sources had complementary strengths and weaknesses; a multi-source surveillance system would be best placed to harness these different strengths. The identified data sources showed diversity in terms of quality but great convergence in relevant variables which will facilitate integration. The study has also revealed limited integration and lack of coordination on the data flow from various sources which may lead to reduced data quality and delay decision making and actions. It is envisaged that integration of the identified data sources by leveraging their strengths while addressing the gaps will be a step towards system improvement - specifically in early warning surveillance. For the integration to be operational, the animal health stakeholders may consider technical, technological, organizational and socio-economic aspects of the surveillance systems. Lastly, coordination among institutions working in animal health surveillance is indispensable to ensure coordinated actions and responses to disease events.

## Supplementary Information

**Additional file 1.**

**Additional file 2.**

**Additional file 3.**

## Data Availability

The datasets used and/or analysed during the current study are available from the corresponding author on reasonable request.

## Abbreviations

ARDS: Agricultural Routine Data System
DVO: District Veterinary Officer
DVS: Director of Veterinary Services
EMA-i: Event Mobile Application
ODK: Open Data Kit
SILAB: *Sistema Informativo di Laboratorio*
TANAPA: Tanzania National Parks
TANLITS: Tanzania National Livestock Identification and Traceability System
TAWIRI: Tanzania Wildlife Research Institute
TVLA: Tanzania Veterinary Laboratory Agency and ZVC- Zonal veterinary centres

## References

[1] Whiting TL (2003). Foreign animal disease outbreaks, the animal welfare implications for Canada : risks apparent from international experience. Can Vet J.

[2] Depa PM, Dimri U, Sharma MC, Tiwari R (2012). Update on epidemiology and control of foot and mouth disease - a menace to international trade and global animal enterprise. Vet World.

[3] Bender JB, Hueston W, Osterholm M (2006). Recent animal disease outbreaks and their impact on human populations. J Agromed.

[4] Evans B (2006). The social and political impact of animal diseases. Vet Ital.

[5] Hoinville L, Ronello A, Alban L. Animal health surveillance terminology final report from pre-ICAHS workshop. Health. 2011:1–26.

[6] Food and Agriculture Organization of the United Nations (2011). Challenges of animal health information systems and surveillance for animal diseases and zoonoses.

[7] Bisdorff B, Schauer B, Taylor N, Rodríguez-Prieto V, Comin A, Brouwer A, Dórea F, Drewe J, Hoinville L, Lindberg A, Aviles MM (2017). Active animal health surveillance in European Union member states: gaps and opportunities. Epidemiol Infect.

[8] Institute of Medicine (US) Committee on a National Surveillance System for Cardiovascular and Select Chronic Diseases. A Nationwide Framework for Surveillance of Cardiovascular and Chronic Lung Diseases. Washington, DC: National Academies Press; 2011. 22259816

[9] Holguín-Veras J. Freight data cost elements (Vol. 22). Washington, D.C. Transportation Research Board; 2013.

[10] Gates MC, Holmstrom LK, Biggers KE, Beckham TR (2015). Integrating novel data streams to support biosurveillance in commercial livestock production systems in developed countries: challenges and opportunities. Front Public Health.

[11] Klompas M, Haney G, Church D, Lazarus R, Hou X, Platt R (2008). Automated identification of acute hepatitis B using electronic medical record data to facilitate public health surveillance. PLoS One.

[12] Dupuy C, Hendrikx P, Hardstaff J, Lindberg A (2012). Contribution of meat inspection to animal health surveillance in bovine animals. EFSA Supporting Publications.

[13] Vial F, Reist M (2014). Evaluation of Swiss slaughterhouse data for integration in a syndromic surveillance system. BMC Vet Res.

[14] Dórea FC, Vial F (2016). Animal health syndromic surveillance: a systematic literature review of the progress in the last 5 years (2011–2016). Vet Med.

[15] Gan WQ, Demers PA, Mcleod CB, Koehoorn M. Population-based asbestosis surveillance in British Columbia. Occup Environ Med. 2009;66(11):766–71. 10.1136/oem.2008.045211 19528044

[16] Bunn TL, Yu L, Ms HAS, Ms MS. Surveillance of methadone-related poisonings in Kentucky using multiple data sources. Pharmacoepidemiol Drug Saf. 2010;19(2):124–31. 10.1002/pds.1901 20077525

[17] Saunders RC, Heflinger CA. Integrating data from multiple public sources: opportunities and challenges for evaluators. Evaluation. 2004;10(3):349–65.

[18] Schnitzer PG, Slusher P, Van Tuinen M (2004). Child maltreatment in Missouri: combining data for public health surveillance. Am J Prev Med.

[19] Dong XL, Saha B, Srivastava D (2012). Less is more: selecting sources wisely for integration. Proc VLDB Endowment.

[20] Kivaria FM, Kapaga AM (2002). Review of current problems and shortcomings in the Tanzanian animal health information system with suggestions on improvement.

[21] Ministry of Livestock and Fisheries, The united Republic of Tanzania (2019). Animal health surveillance strategy.

[22] National bureau of statistics - population and housing census. [cited 2020 Sep 17]. Available from: https://www.nbs.go.tz/index.php/en/census-surveys/population-and-housing-census

[23] The United Republic of Tanzania (URT) (2016). Kongwa District Social-Economic profile.

[24] The United Republic of Tanzania (URT) (2017). Kibaha district council Investment profile.

[25] QGIS (2020). Development team.

[26] Bengtsson M (2016). How to plan and perform a qualitative study using content analysis. NursingPlus Open.

[27] Erlingsson C, Brysiewicz P (2017). A hands-on guide to doing content analysis. Afr J Emerg Med.

[28] TechnoHealth Surveillance. Towards AfyaData-EMA-i-SILAB interoperability for improved surveillance. 2019;4(3). [ cited 2020 June 2020] Available from http://www.sacids.org/wp-content/uploads/2019/07/newsletter.pdf

[29] Karimuribo ED, Mutagahywa E, Sindato C, Mboera L, Mwabukusi M, Kariuki Njenga M (2017). A smartphone app (Afyadata) for innovative one health disease surveillance from community to national levels in africa: intervention in disease surveillance. JMIR Public Health Surveill.

[30] FAO (2015). EMA-i : A mobile app for timely animal disease field reporting to enhance surveillance.

[31] Motta P, Porphyre T, Handel I, Hamman SM, Ngu Ngwa V, Morgan KL, Tanya VN, Bronsvoort BM (2019). Characterizing livestock markets, primary diseases and key management practices along the livestock supply chain in Cameroon. Front Vet Sci.

[32] Rautureau S, Dufour B, Durand B (2011). Vulnerability of animal trade networks to the spread of infectious diseases: a methodological approach applied to evaluation and emergency control strategies in cattle, France, 2005. Transbound Emerg Dis.

[33] Onono JO, Wieland B, Rushton J (2013). Factors influencing choice of veterinary service provider by pastoralist in Kenya. Trop Anim Health Prod.

[34] Van Den Bossche BP, Thys E, Elyn R, Marcotty T, Geerts S (2004). The provision of animal health care to smallholders in Africa: an analytical approach: -EN- -FR- -ES. Rev Sci Tech OIE.

[35] Shaffer JG, Doumbia S, Ndiaye D, Diarra A, Gomis JF, Nwakanma D, et al. Development of a data collection and management system in West Africa: challenges and sustainability. Infect Dis Poverty. 2018;7(1):125. 10.1186/s40249-018-0494-4 PMC6292095 30541626

[36] Integrated disease surveillance and response technical guidelines, booklet one: introduction section [Internet]. WHO | Regional Office for Africa. [cited 2020 Sep 17]. Available from: https://www.afro.who.int/publications/integrated-disease-surveillance-and-response-technical-guidelines-booklet-one

[37] European Centre for Disease Prevention and Control (2014). Data quality monitoring and surveillance system evaluation: a handbook of methods and applications.

[38] Falzon LC, Alumasa L, Amanya F, Kang’ethe E, Kariuki S, Momanyi K (2019). One health in action: operational aspects of an integrated surveillance system for zoonoses in western Kenya. Front Vet Sci.

[39] Gilbert WH, Häsler BN, Rushton J (2014). Influences of farmer and veterinarian behaviour on emerging disease surveillance in England and Wales. Epidemiol Infect.

[40] Peeler E (2019). Disease reporting is fundamental to aquatic animal health.

[41] Keusch G, Institute of Medicine (U.S.) (2009). Sustaining global surveillance and response to emerging zoonotic diseases.

[42] Wolkin AF, Patel M, Watson W, Belson M, Rubin C, Schier J (2006). Early detection of illness associated with poisonings of public health significance. Ann Emerg Med.

[43] Ahmed K, Temate Y, Tiagueu JA, Stover DL, Peters PJ, Brooks JT, Sangareddy SP, Dcruz JJ. Integrating data from disparate data systems for improved HIV reporting: Lessons learned. Online J Public Health Informatics. 2018;10(1):e49

[44] Tanzania communications regulatory authority. Quarterly Communications Statistics. [cited 2020 Sep 17]. Available from https://tcra.go.tz/publication-and-statistics/statistics

[45] UNDP. Capacity development: A UNDP primer [Internet]. [cited 2020 Sep 17]. Available from: https://www.undp.org/content/undp/en/home/librarypage/capacity-building/capacity-development-a-undp-primer.html

[46] Toutant S, Gosselin P, Bélanger D, Bustinza R, Rivest S (2011). An open source web application for the surveillance and prevention of the impacts on public health of extreme meteorological events: the SUPREME system. Int J Health Geogr.

[47] Kshirsagar DP, Savalia CV, Kalyani IH, Kumar R, Nayak DN. Disease alerts and forecasting of zoonotic diseases: an overview. Vet World. 2013;6(11):889–96

[48] George J, Häsler B, Mremi I, Sindato C, Mboera L, Rweyemamu M, Mlangwa J (2020). A systematic review on integration mechanisms in human and animal health surveillance systems with a view to addressing global health security threats. One Health Outlook.

