# Supplementary material for: Towards an integrated animal health surveillance system in Tanzania: making better use of existing and potential data sources for early warning surveillance
Source: BMC Vet Res. 2021 Mar 6;17:109. doi: 10.1186/s12917-021-02789-x (PMC7936506; doi:10.1186/s12917-021-02789-x)
Supplement: Supplementary file 1 — Additional file 1. [file 12917_2021_2789_MOESM1_ESM.docx]

**Table 1: Type of records available in different data sources**

|  | **Records Type** | | | | | | | | | | |
| --- | --- | --- | --- | --- | --- | --- | --- | --- | --- | --- | --- |
| **data source** | **Name of the place** | **Type of the animal** | **Georeference** | **Case** | **Syndromes** | **Photos** | **Number of affected** | **Number of recovered** | **Number of deaths** | **Number of treated** | **Medication** |
| **Livestock farmers** | Yes | Yes | No | No | Yes | No | Yes | Yes | Yes | Yes | Yes |
| **Commercial livestock farms** | Yes | Yes | No | No | Yes | No | Yes | Yes | Yes | Yes | Yes |
| **Livestock markets** | Yes | Yes | No | No | Yes | No | Yes | No | No | No | No |
| **Animal dip sites** | Yes | Yes | No | No | No | No | No | No | No | No | No |
| **Slaughter facilities** | Yes | Yes | No | Yes | Yes | No | Yes | No | No | No | No |
| **Zoo-sanitary checkpoints** | Yes | Yes | No | No | Yes | No | Yes | No | No | No | No |
| **Veterinary shops** | Yes | Yes | No | No | Yes | No | No | No | No | No | Yes |
| **AfyaData** | Yes | Yes | Yes | No | Yes | Yes | Yes | Yes | Yes | No | No |
| **Agricultural routine data system (ARDS)** | Yes | Yes | No | Yes | Yes | No | Yes | Yes | Yes | Yes | Yes |
| **SILAB** | Yes | Yes | No | Yes | No | No | No | No | No | No | No |
| **EMA-i** | Yes | Yes | Yes | Yes | Yes | Yes | Yes | Yes | Yes | No | No |
| **TANLITS** | Yes | Yes | Yes | No | No | No | No | No | No | No | No |
| **TAWIRI** | Yes | Yes | No | Yes | Yes | No | Yes | Yes | Yes | No | No |
