# Supplementary material for: Towards an integrated animal health surveillance system in Tanzania: making better use of existing and potential data sources for early warning surveillance
Source: BMC Vet Res. 2021 Mar 6;17:109. doi: 10.1186/s12917-021-02789-x (PMC7936506; doi:10.1186/s12917-021-02789-x)
Supplement: Supplementary file 2 — Additional file 2. [file 12917_2021_2789_MOESM2_ESM.docx]

**Supplementary 1: Questionnaire for government officials**

**Date of the interview:………………………………**

**Starting time:…………………………………….**

**Finishing time:……………………………….**

1. **Region**
2. Arusha
3. Pwani
4. Dodoma
5. **Districts**
6. Kibaha
7. Kongwa
8. Ngorongoro
9. **Ward (On the separate list)**

**Section A: Demographic characteristics of the respondent**

1. **Respondents’ ID:………………………………**
2. **Respondent’s designation:………………………………………. (tick only one)**
3. Livestock field officer
4. Ranch manager
5. District Veterinary Officer
6. ZVC Manager
7. TVLA Manager
8. Ministerial official
9. Others…mention
10. **Age…………………………**
11. **Education level**
12. Primary school
13. Secondary School
14. Certificate level
15. Diploma
16. Degree
17. Higher degrees
18. **Gender**

**0.** Male

1. Female

1. Years of experience in the field……..
2. Years of experience in the current position….

**Section B: Implementation of surveillance activities**

1. **What is your area coverage?**
2. One village
3. Two villages
4. Three village
5. More than three village
6. **The closest site/village is how many kms from your work station?**
7. **The furthest site/village is how many kms from your work?**
8. What are your roles in surveillance activities? (tick all that are relevant)
   1. Collecting data from the primary sources
   2. Data compilation and integration
   3. Data quality assurance
   4. Database management
   5. Data analysis and interpretation
   6. Dissemination
   7. Response
9. **What are your sources of surveillance information (Tick all mentioned)**
   1. Livestock keepers
   2. Slaughter facilities
   3. Veterinary centers/clinics/facilities
   4. Vetshops
   5. Livestock markets
   6. Zoo sanitary checkpoints
   7. Milk collection centres
   8. Livestock field officers
   9. DVOs
   10. ZVCs
   11. Diagnostic facilities
   12. Others….mentioned
10. **How frequent do you collect data from the mentioned sources**

| **Source** | **Frequency (choose appropriate answer)**  Daily (1), Weekly (2), Bi-weekly (3), Monthly(4), Upon occurrence of suspected cases (5),Not applicable(0) |
| --- | --- |
| Livestock keepers |  |
| Slaughter facilities |  |
| Veterinary centers/clinics/facilities |  |
| Vetshops |  |
| Livestock markets |  |
| Zoosanitary checkpoints |  |
| Milk collection centres |  |
| Livestock field officers |  |
| DVOs |  |
| ZVCs |  |
| Diagnostic facilities |  |
| Others |  |

1. **Which tools do you use to surveillance data? (Tick all mentioned)**
   1. Field surveillance forms (Paper-based)
   2. Digitized surveillance forms (E-Mai)
   3. Digitized surveillance forms (Afyadata)
   4. Others…mentioned
2. **How do you transmit surveillance information? (Tick all mentioned)**
3. Transporting them to the respective authority
4. Electronic data transmission (real-time)
5. Electronic data transmission (emails)
6. Making phone calls to the respective authorities
7. **For the last six months, how many times did you transmit data by;**

| **Transmission mode** | **Frequency** |
| --- | --- |
| Transporting them to the respective authority | (trips) |
| Electronic data transmission (real-time) | (number of forms sent) |
| Electronic data transmission (emails) | (Number of emails sent) |
| Making phone calls to the respective authorities | (number of phone calls made) |

1. **How do you compile surveillance data from the source/lower level?**
   1. Manually from the sources
   2. Real-time compilation into central repository (electronic)
   3. I don’t compile
2. **How often do you compile data from the lower levels?**
3. Real-time
4. Daily
5. Weekly
6. Monthly
7. More than a month
8. **How do you store surveillance data**
9. Physical files
10. Electronic file system
11. Database
12. **How often do you check data for quality assurance and cleaning? (*Ask this from DVOs and higher)***
13. Real-time
14. Daily
15. Weekly
16. Monthly
17. More than a month
18. **How often do you do analysis and interpretation of surveillance data? (*For those responsible for this role)***
19. Real-time
20. Daily
21. Weekly
22. Monthly
23. Quarterly a year
24. Semi-annually
25. Annually
26. When need be
27. Never
28. **When was the last time you did the analysis and interpretation?**
29. In the last 24hours
30. Within this week
31. Last week
32. Last month
33. Last quarter
34. In the last six month
35. Last year
36. Never
37. **Who are the recipient of the surveillance information that you collect?**
38. Livestock field officer
39. Ranch manager
40. District Veterinary Officer
41. ZVC Manager
42. TVLA Manager
43. DVS
44. International organizations
45. Business people
46. Others….mention
47. **Do you normally share surveillance information with officers/actors in other sectors? (1. Yes 0. No)**

If yes, who are they?….mention

1. **What are the communication channels do you use to communicate surveillance information to stakeholders?**

| **Channel** | **F Frequency (choose appropriate answer)**  Real-time (1), Daily (2), Weekly (3), Monthly (4) , Quarterly a year (5), Semi-annually (6), Annually (7), When need be (8), Never(0) |
| --- | --- |
| Real time reports through mobile technologies |  |
| Telephone calls |  |
| Case reports |  |
| Monthly reports |  |
| Quarterly surveillance bulletin |  |
| Formal meetings |  |
| Word of mouth |  |
| Press release |  |
