# Supplementary material for: Towards an integrated animal health surveillance system in Tanzania: making better use of existing and potential data sources for early warning surveillance
Source: BMC Vet Res. 2021 Mar 6;17:109. doi: 10.1186/s12917-021-02789-x (PMC7936506; doi:10.1186/s12917-021-02789-x)
Supplement: Supplementary file 3 — Additional file 3. [file 12917_2021_2789_MOESM3_ESM.docx]

**Supplementary 2: Questionnaire for Veterinary shop owners/shopkeepers**

**Date of the interview:………………………………**

**Starting time:…………………………………….**

**Finishing time:……………………………….**

1. **Region**
2. Arusha
3. Pwani
4. Dodoma
5. **Districts**
6. Kibaha
7. Kongwa
8. Ngorongoro
9. **Ward (On the separate list)**
10. Name of the shop
11. Shop ID
12. Average number of customers saved per week
13. Is there any information collected before disbursing the medicine?

(i)Yes (ii) No

1. Which information do you collect before disbursing medicine?............................................
2. What do you do with the information you collect from the client?
3. How do you store sales information?

1. Manual/paper

2. Computer

3. Phone
